# A Novel Marine Pyran-Isoindolone Compound Enhances Fibrin Lysis Mediated by Single-Chain Urokinase-Type Plasminogen Activator

**DOI:** 10.3390/md20080495

**Published:** 2022-07-30

**Authors:** Chunli Gao, Simin Tang, Haixing Zhang, Huishu Zhang, Tian Zhang, Bin Bao, Yuping Zhu, Wenhui Wu

**Affiliations:** 1 Department of Marine Bio-Pharmacology, College of Food Science and Technology, Shanghai Ocean University, Shanghai 201306, China; chunlgao@163.com (C.G.); tang_sm414@163.com (S.T.); 18029731134@163.com (H.Z.); hpzhanghuishu2022@163.com (H.Z.); tianzhang199800@163.com (T.Z.); bbao@shou.edu.cn (B.B.); 2 Shanghai Engineering Research Center of Aquatic-Product Processing and Preservation, Shanghai 201306, China; 3 Basic Medical Experimental Teaching Center, Basic Medical College, Naval Medical University, PLA, Shanghai 200433, China

**Keywords:** FGFC1, plasminogen, fibrinolytic activity, fibrin clot, fibrinolysis

## Abstract

Fungi fibrinolytic compound 1 (FGFC1) is a rare pyran-isoindolone derivative with fibrinolytic activity. The aim of this study was to further determine the effect of FGFC1 on fibrin clots lysis in vitro. We constructed a fibrinolytic system containing single-chain urokinase-type plasminogen activator (scu-PA) and plasminogen to measure the fibrinolytic activity of FGFC1 using the chromogenic substrate method. After FITC-fibrin was incubated with increasing concentrations of FGFC1, the changes in the fluorescence intensity and D-dimer in the lysate were measured using a fluorescence microplate reader. The fibrin clot structure induced by FGFC1 was observed and analyzed using a scanning electron microscope and laser confocal microscope. We found that the chromogenic reaction rate of the mixture system increased from (15.9 ± 1.51) × 10^−3^ min^−1^ in the control group to (29.7 ± 1.25) × 10^−3^ min^−1^ for 12.8 μM FGFC1(*p* < 0.01). FGFC1 also significantly increased the fluorescence intensity and d-dimer concentration in FITC fibrin lysate. Image analysis showed that FGFC1 significantly reduced the fiber density and increased the fiber diameter and the distance between protofibrils. These results show that FGFC1 can effectively promote fibrin lysis in vitro and may represent a novel candidate agent for thrombolytic therapy.

## 1. Introduction

Cardiovascular diseases (CVDs) have become a rapidly emerging health problem in the modern world because of their high incidence rate, high disability rate, and high mortality rate, and they are one of the leading causes of death around the world [1]. It has been reported that intravascular thrombosis is the main cause of CVDs, including atherosclerotic heart disease, cerebrovascular disease, and venous thromboembolism [2]. It is estimated that approximately 18 million people die from CVDs every year, and the mortality rate is close to that of cancer [3,4], seriously threatening human health. In addition, CVDs are associated with increased healthcare costs, resulting in a huge economic burden to human beings. According to statistics, the average annual cost of CVD and stroke in the United States was estimated to be about USD 351 billion from 2014 to 2015 [5], with the European Union spending EUR 169 billion on CVD ever year [6]. Therefore, thrombolysis and blood flow reconstruction are very important for the treatment of thrombotic diseases.

Fibrinogen, as the precursor of fibrin, is converted to fibrin by thrombin [7,8]. Fibrin is the main component of thrombus [9]. Cross-linked fibrin polymers (fibrin clots) are essential for hemostasis and the formation of pathological thrombus [10,11]. Generally, there is a dynamic balance between fibrin formation (coagulation) and fibrinolysis in vivo to maintain the patency of blood vessels [12,13]. Any disruption to this equilibrium may result in thrombosis or hemorrhage, that is, the excessive activation of coagulation may cause the development of thrombi, while excessive fibrinolysis may lead to severe bleeding [14].

Fibrinolysis is a method that contributes the prevention of fibrin clot formation [15,16]. The fibrinolytic system plays an important role in fibrinolysis and is mainly composed of plasminogen, fibrinogen, and plasminogen activators (such as the tissue-type plasminogen activator and urokinase-type plasminogen activator) and their inhibitors (including plasminogen activator inhibitor type 1, plasminogen activator inhibitor type 2 and protein C inhibitor) [17,18]. The activation of the fibrinolytic system destroys the stability of the thrombus structure and help to restore intravascular blood flow [19]. Most thrombolytic agents are plasminogen activators, which convert plasminogen into active plasmin by specific cleavage of the peptide bond between Arg561 and Val562, resulting in the degradation of fibrin. Additionally, there are plasmin-like proteins directly acting on fibrinogen or fibrin to degrade blood clots such as alfimeprase and microplasmin [20,21]. At present, fibrinolytic drugs approved by the United States Food and Drug Administration (FDA) mainly include streptokinase, urokinase, alteplase, tenecteplase, etc. [22,23,24,25]. However, these thrombolytic agents have some undesirable defects, including high price, limited specificity towards fibrin, a short half-life, serious hemorrhagic risks, and allergic reactions [22,26,27,28]. In view of these limitations, searching for a safe, efficient, cheap, and novel fibrinolytic drug is of tremendous clinical value.

Marine microorganisms can produce a variety of novel metabolites, important resources for the discovery of new bioactive compounds [29]. There is growing evidence that active fibrinolytic products that have been isolated from marine microorganisms can improve pharmacological properties and show minimal side effects [30,31,32]. Among them, small-molecule natural products with fibrinolytic activity play a fibrinolytic role by regulating the pro-enzymes in the fibrinolytic system without affecting the catalytic activity of the mature enzymes and have attracted increasing attention due to their great potential and safety in thrombolysis [33,34]. For example, Shinohara et al. isolated staplabin, a novel triprenyl phenol compound from a culture of *Stachybotrys microspora*, as an active component that was able to stimulate the binding of plasminogen to U937cells and fibrin [35].

**Figure 1:**
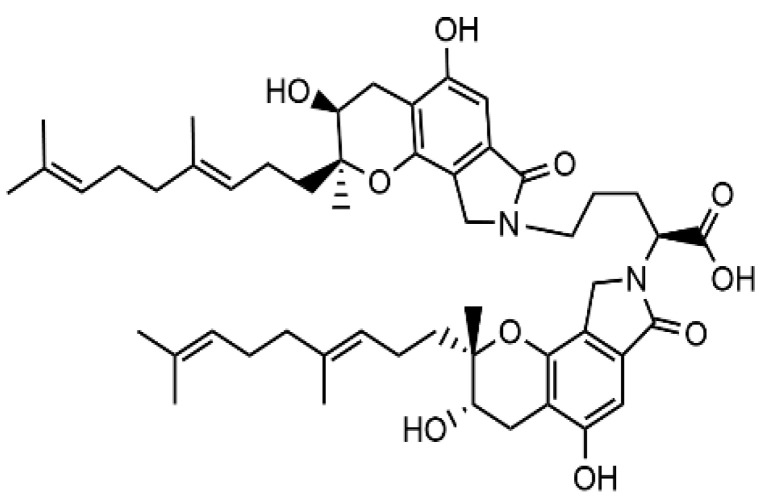
The structure of FGFC1.

In our previous studies, we isolated a pyran-isoindolone compound FGFC1 (Figure 1) with a molecular weight of 869 Da from a rare marine microorganism *Stachbotrys longispora* FG216, and characterized its fibrinolytic activity [36,37,38]. Based on these results, this study aims to further investigate the fibrinolytic activity of FGFC1 and its effect on fibrin network lysis in vitro.

## 2. Results

### 2.1. Fibrinolytic Activity of FGFC1

**Figure 2:**
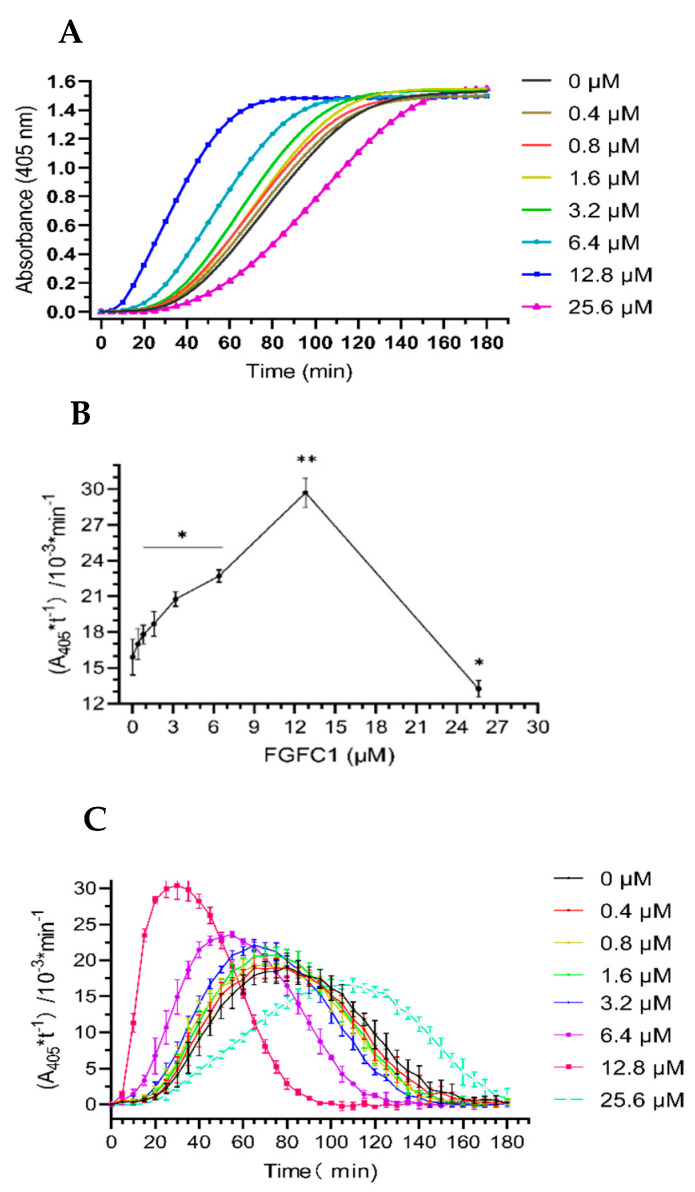
Fibrinolytic activity of FGFC1 (0–25.6 μM). (**A**) The absorbance–time curve of the mixture system was measured at 405 nm at 37 °C for 180 min using a microplate reader. (**B**) The Kn (the slope of each curve) values of FGFC1 (0–25.6 μM) in the reaction system. Results expressed as the mean ± SD of three parallel experiments. * *p* < 0.05, ** *p* < 0.01, compared to the control group (absence FGFC1). (**C**) The reaction rates of FGFC1 (0–25.6 μM) at different times (0–180 min).

The fibrinolytic activity of increasing concentrations of FGFC1 was evaluated using the urokinase chromogenic substrate method shown in Figure 2. It can be seen from Figure 2A that the value of A405 increased linearly over time in the early stage of the enzymatic reaction. The FGFC1 concentration increased from 0.4 μM to 12.8 μM, and when the FGFC1 concentration was higher, the reaction rate of the mixture system was greater (that is the fibrinolytic activity was stronger). The data in Figure 2A,B show that the effects of FGFC1 (0.4–25.6 μM) on plasminogen include activation and inhibition. FGFC1 (0.4–12.8 μM) promoted the reaction rate of the mixture system in a concentration-dependent manner, increasing from (15.9 ± 1.51) × 10^−3^ min^−1^ in the control group to (29.7 ± 1.25) × 10^−3^ min^−1^ for 12.8 μM FGFC1 (*p* < 0.01), and enhancement of about 87%. However, when the FGFC1 concentration was 25.6 μM, the reaction rate was significantly inhibited compared to that in the absence of FGFC1, which decreased from (15.9 ± 1.51) × 10^−3^ min^−1^ to (13.2 ± 0.71) × 10^−3^ min^−1^ (*p* < 0.05), resulting in a decrease of about 17%.

From 0.4 μM to 12.8 μM, FGFC1 shortened the time to reach the maximum reaction rate in a concentration-dependent manner. The time for 0–3.2 μM, 6.4 μM, and 12.8 μM FGFC1 to achieve the maximum reaction rate was between 60 and 80 min, 45 and 60 min, and 20 and 40 min, respectively, while 25.6 μM FGFC1 showed an inhibitory effect and reached the maximum reaction rate between 90 and 120 min (Figure 2A–C). These data show that a certain FGFC1 concentration can significantly improve the fibrinolytic activity mediated by plasminogen and scu-PA, suggesting that FGFC1 has a good fibrinolytic effect. According to the results of this experiment, we chose 0.4–12.8 μM FGFC1 to further study its effect on fibrin lysis and structure.

### 2.2. FGFC1 Promote FITC-Fibrin Lysis In Vitro

Fibrinogen was labeled with FITC and then converted to FITC-fibrin by injecting 10 U/mL thrombin and 0.1% calcium chloride. Subsequently, FITC-fibrin was added to the mixture system containing plasminogen and scu-PA to evaluate the effect of FGFC1 on fibrinolysis. This reaction system simulates the physiological environment of fibrinolysis in vivo. Therefore, in this reaction system, the impact of FGFC1 on FITC-fibrin lysis can be determined by measuring the changes in the fluorescence intensity and d-dimer concentration.

**Figure 3:**
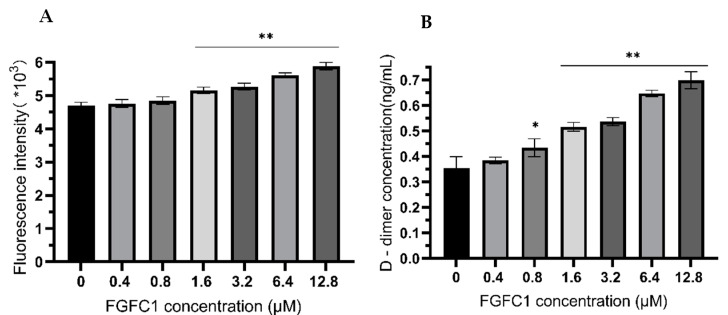
FGFC1 (0–12.8 μM) promotes fibrinolysis in vitro. (**A**) Fluorescence intensity of the mixture system was measured at the excitation wavelength of 495 nm and the emission wavelength of 520 nm after incubation at 37 °C for 20 min. (**B**) Enzyme-linked immunosorbent assay was used to detect effect of FGFC1 (0–12.8 μM) on lysis of FITC-fibrin. Results expressed by the mean ± SD of three parallel experiments. * *p* < 0.05, ** *p* < 0.01, compared to the control group (absence FGFC1).

The dissolution of FITC-fibrin was analyzed by fluorescence measurements after incubation both without and with increasing concentrations of FGFC1. The results showed that FGFC1 increased the fluorescence intensity in a concentration-dependent manner. The fluorescence intensities of 0.4 μM and 0.8 μM FGFC1 were (4.76 ± 0.12) × 10^3^ and (4.85 ± 0.11) × 10^3^, respectively, with no differences being observed compared to the control group (4.69 ± 0.10) × 10^3^. However, the fluorescence intensity increased significantly when the FGFC1 concentrations increased from 1.6 μM to 12.8 μM, increasing from (4.69 ± 0.10) × 10^3^ for vehicle (in the absence of FGFC1) to (5.15 ± 0.10) × 10^3^, (5.26 ± 0.10) × 10^3^, (5.61 ± 0.07) × 10^3^, and (5.88 ± 0.11) × 10^3^ for 1.6 μM, 3.2 μM, 6.4 μM, and 12.8 μM FGFC1, respectively (Figure 3A).

The D-dimer content was estimated by ELISA incubation both with and without FGFC1. FGFC1 increased the concentration of d-dimer in a dose-dependent manner, showing a onefold enhancement from 0.35 ± 0.04 ng/mL for vehicle to 0.70 ± 0.03 ng/mL for 12.8 μM FGFC1 (Figure 3B). These results showed that FGFC1 could effectively promote the lysis of FITC-fibrin in vitro.

### 2.3. FGFC1 Effect on Fibrin Fiber Nanostructure by Scanning Electron Microscopy

**Figure 4:**
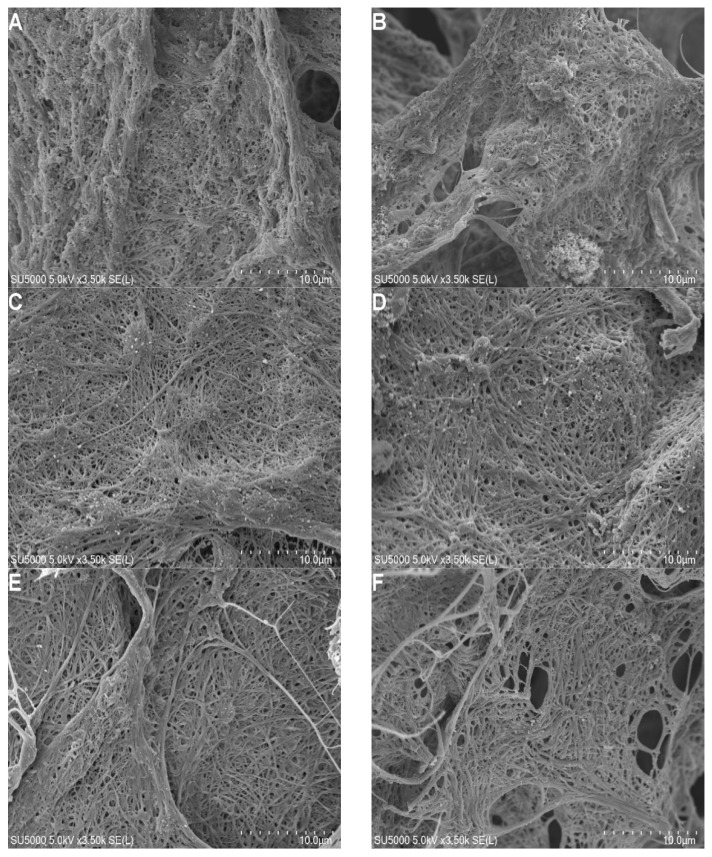

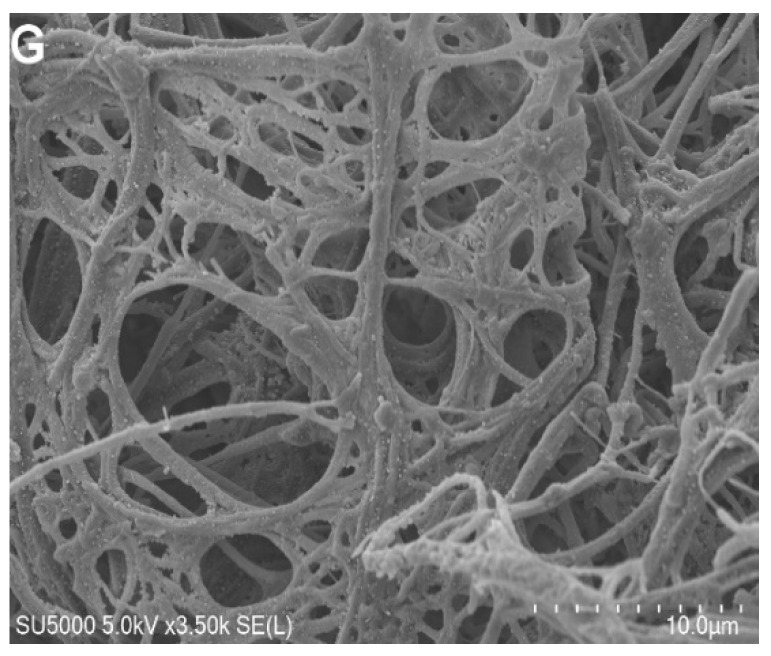
Scanning electron microscopy images of fibrin clots (all scale bars are 10 μm). Clotting was initiated in the (**A**) absence and (**B**–**G**) presence (0.4–12.8 μM) of FGFC1 by 1 U/mL thrombin and 15 mM CaCl_2_.

**Figure 5:**
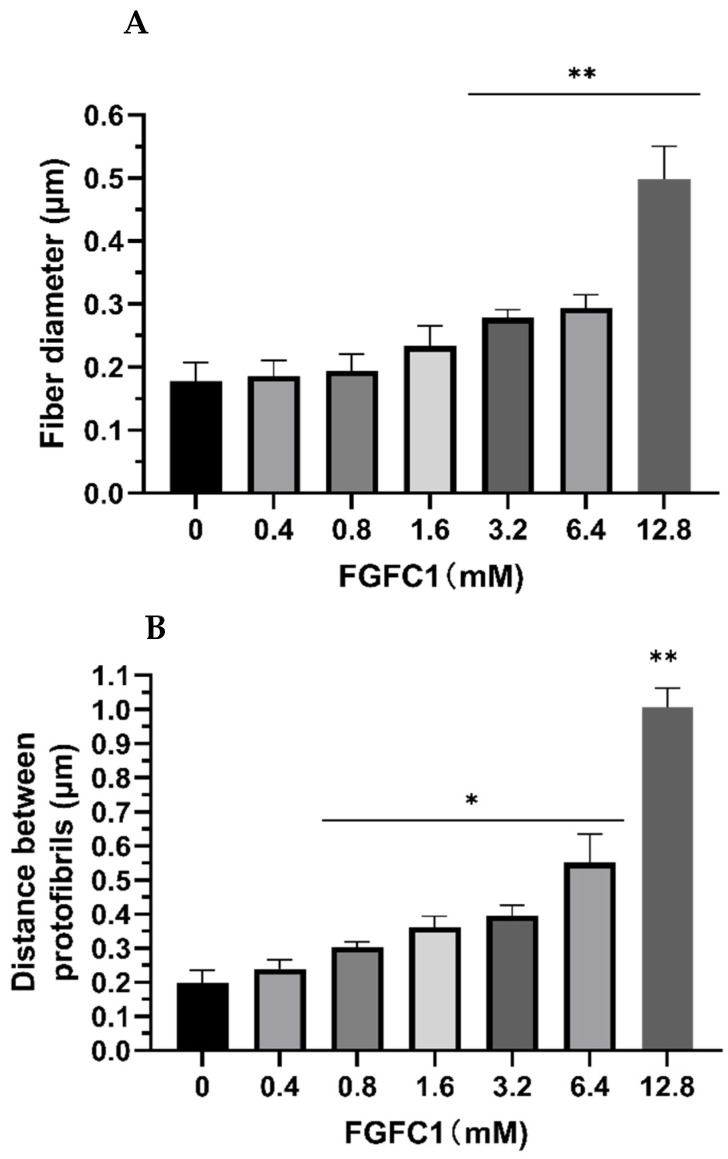

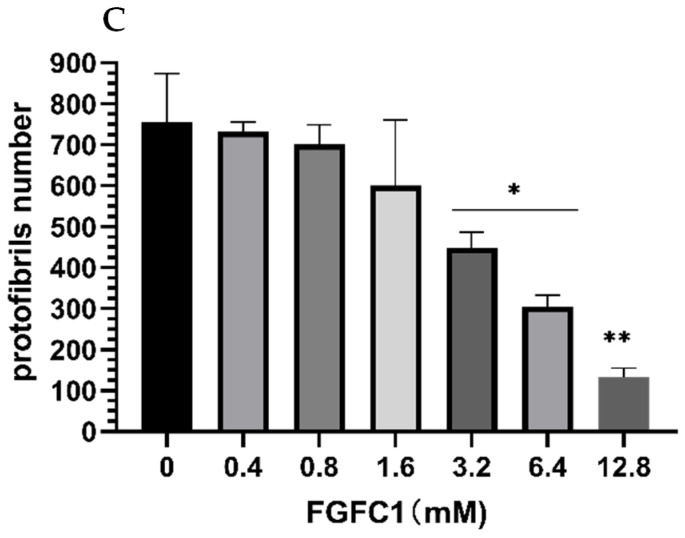
Effect of FGFC1 (0–12.8 μM) on the molecular structure of fibrin fibers (scanning electron microscopy). (**A**) Diameter of fibrin fiber. (**B**) Distance between protofibrils within fibrin fibers. (**C**) Number of protofibrils. Results expressed by the mean ± SD of three parallel experiments. * *p* < 0.05, ** *p* < 0.01, compared to the control group (absence FGFC1).

The changes in the fibrin structure during the degradation process induced by increasing the FGFC1 concentration using scanning electron microscopy in the dried condition are shown in Figure 4. Fibrin clots in the absence of FGFC1 showed thicker and more densely knit fibers that were interspaced with smaller pores. In contrast, an increase in the FGFC1 concentration resulted in significant differences in the density, diameter, and distance of the fibrin fibers. The average fiber diameter increased from 0.18 ± 0.03 μm for the control group to 0.50 ± 0.05 μm for 12.8 μM FGFC1 (an increase of about 1.8-fold, *p* < 0.01, Figure 5A) together with an increase in the distance between the protofibrils from 0.20 ± 0.04 μm to 1.01 ± 0.06 μm (an increase of about 4.1-fold, *p* < 0.01, Figure 5B), while the number of protofibrils decreased from 755 ± 119 to 113 ± 22 (a decrease of about 82%, *p* < 0.01, Figure 5B). These results show the impact of FGFC1 on the structure of the fibrin network.

### 2.4. Images of Fibrin Networks by Confocal Laser Scanning Microscopy

**Figure 6:**
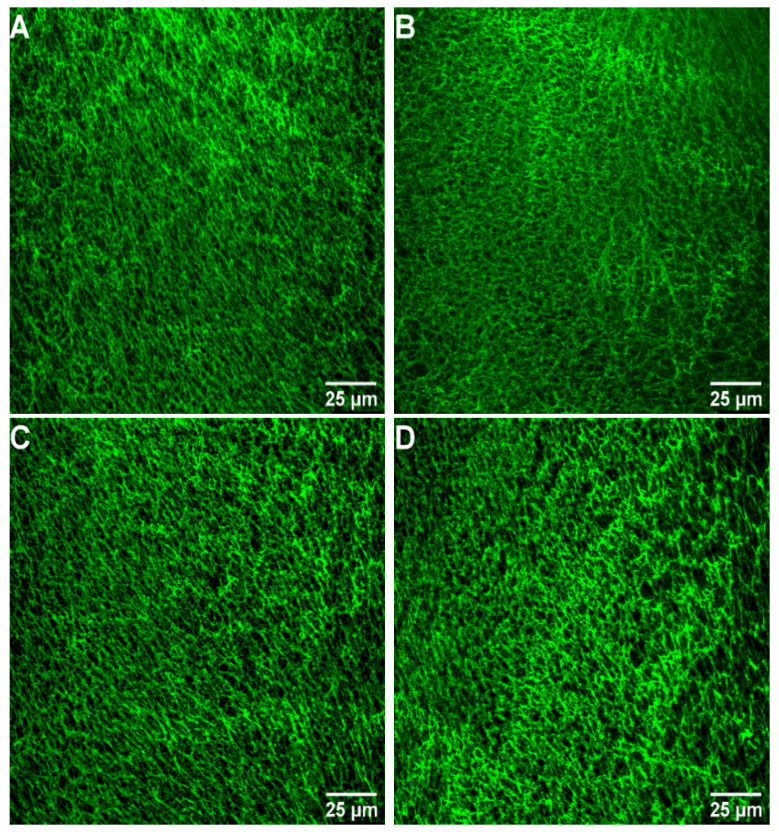

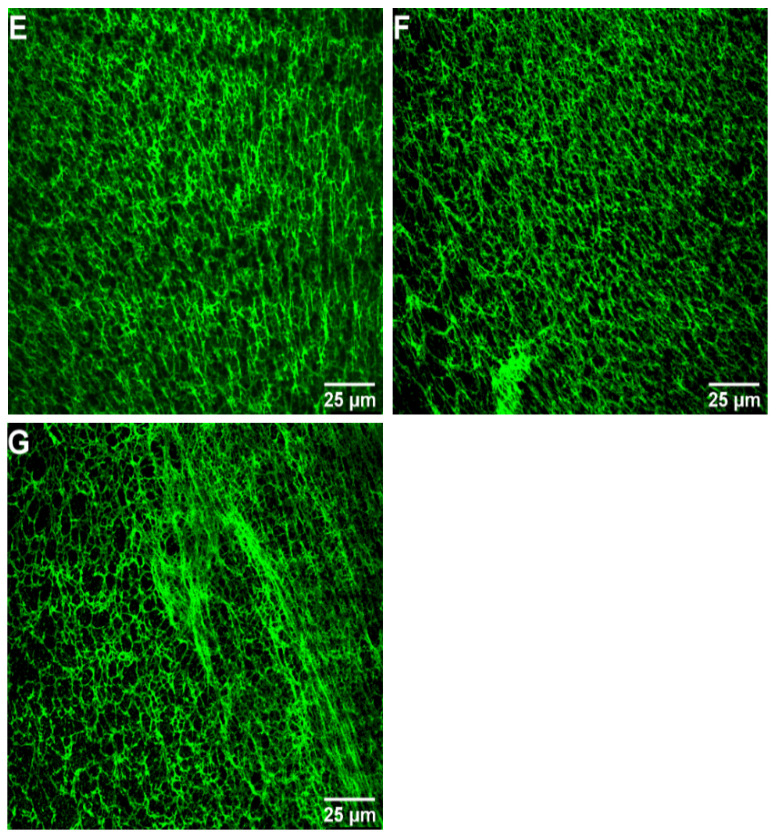
Confocal laser scanning microscopy images of fibrin networks (all scale bars are 25 μm). Clotting was initiated in the (**A**) absence and (**B**–**G**) presence (0.4–12.8 μM) of FGFC1 by 0.6 U/mL thrombin and 8 mM CaCl_2_. Fibrin networks were stained with FITC.

**Figure 7:**
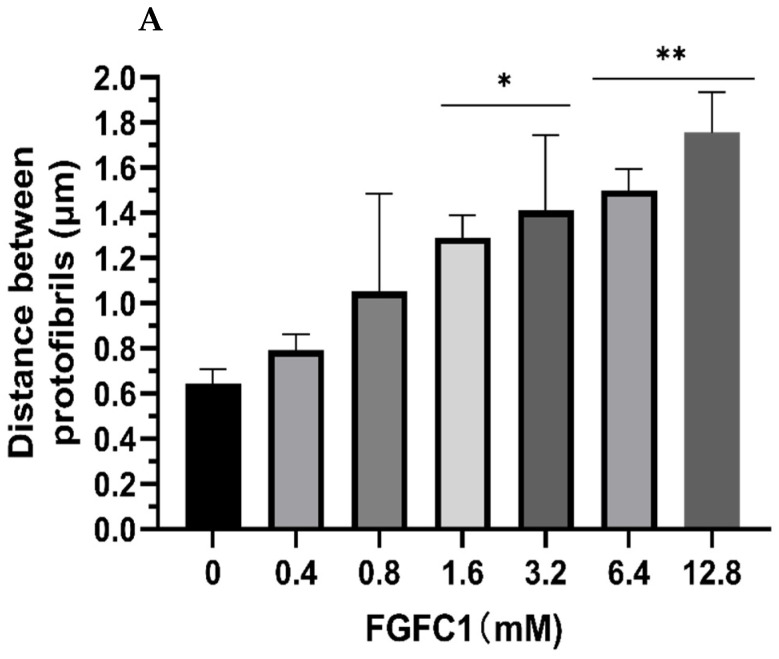

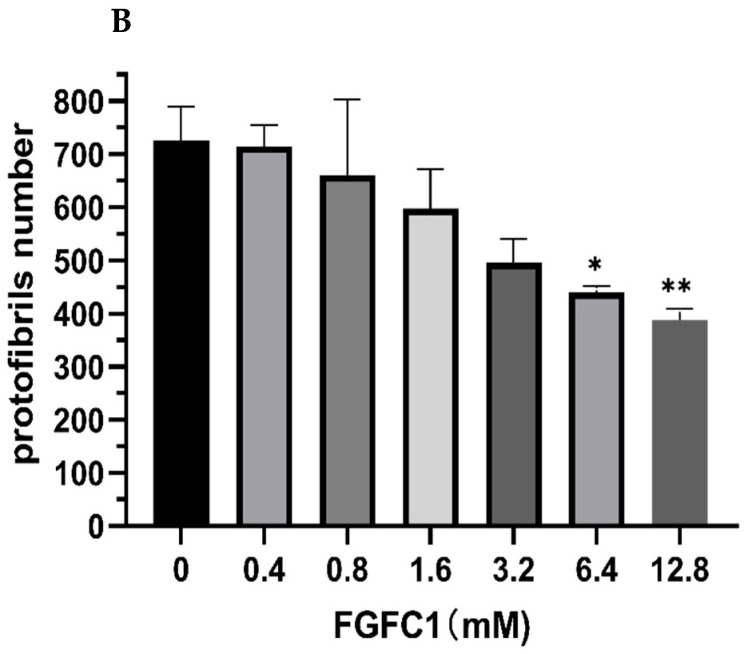
Effect of FGFC1 (0–12.8 μM) on the molecular structure of fibrin fibers by laser scanning confocal microscopy. (**A**) Distance between protofibrils within fibrin fibers. (**B**) Number of protofibrils. Results expressed by the mean ± SD; *n* = 3. * *p* < 0.05, ** *p* < 0.01, compared to the control group (absence of FGFC1).

Laser scanning confocal microscopy was used to further analyze the effects of FGFC1 (0–12.8 μM) on the fibrin network structure in wet conditions. It can be seen from Figure 6 that fibrin maintained a physiological structure in the absence of FGFC1, while increasing FGFC1 concentrations led to the formation of a more porous network structure that was aggregated by thinner fibrin fibers. The distance between protofibrils increased from 0.64 ± 0.06 μm in the control group to 1.76 ± 0.18 μm for 12.8 μM FGFC1 (increased about1.7-fold, *p* < 0.01, Figure 7A), while the number of protofibrils decreased from 726 ± 63 to 389 ± 20 (a decrease of about 46%, *p* < 0.01, Figure 7B).

## 3. Discussion

Most of the common thrombolytic drugs that are used in clinic, such as streptokinase, tissue-type plasminogen activator (tPA), and uPA, are macromolecular compounds, while small molecular compounds have not been used to treat thrombotic diseases [39]. In the fibrinolytic system, the activation of zymogen is an allosteric process. A large number of studies have shown that small molecular compounds with fibrinolytic activity are allosteric effectors in the process of zymogen activation [40,41]. Selecting the inhibitors, antagonists, or activators of enzymes is an effective pharmacological means for the treatment of various diseases. As pro-enzyme regulators, small molecule compounds play a role depending on when and where the enzyme is prompted to work in the physiological system [39]. Therefore, the study of small molecular fibrinolytic compounds will contribute to the exploitation of new drugs to treat thrombotic diseases. Our previous studies have shown that FGFC1 is a rare marine Bisindole alkaloid fibrinolytic compound with isoindolylopyran as its mother nucleus connecting isoamyl alkene chain and valerate group, which is completely different from bacterial and fungicidal fibrinolytic enzymes such as nattokinase(NK), *boletus pseudocalopus* fibrinolytic enzyme (BPFE) [42,43,44,45,46]. NK is a single-chain polypeptide serine protease composed of 275 amino acid residues with a molecular weight of 27.7 kDa [47]. NK regulates fibrinolysis directly or indirectly through the following mechanisms: NK directly degrades fibrin into small-molecular peptides and amino acids; activating the conversion of pro-urokinase to urokinase; inactivating PAI-1 to enhance fibrinolysis [47,48]. However, NK is a protease sensitive to gastric acid. If it is taken orally, the purpose of thrombolysis cannot be achieved [47]. BPFE is purified from fruiting bodies of wild-growing mushroom Boletus pseudocalopus Hongo, composed of amino acid residues with a molecular weight of 63.5 kDa, while there are no studies that have evaluated the fibrinolytic mechanism of BPFE [46]. FGFC1 does not have biological enzyme activity and that the key target of it in terms of its thrombolytic effect is plasminogen. FGFC1 binds to the lysine-binding sites of plasminogen and activates plasminogen to form an open conformation, which is easily activated by plasminogen activator, thereby improving fibrinolytic activity [4,45]. Therefore, we think that FGFC1 can be used as a new small-molecule thrombolytic drug candidate. In this study, we constructed a fibrinolytic system in vitro to further study the effect of FGFC1 on fibrin clot lysis. The results showed that FGFC1was able to increase the uPA-mediated cleavage of the substrate S-2444 in vitro and could effectively promote the fibrin degradation.

It is well known that scu-PA with weak intrinsic activity can cleave the peptide bond between Arg561-Val562 in plasminogen and convert it into a small amount of active plasmin. Next, this small amount of active plasmin promotes the conversion of scu-PA into the two-chain urokinase-type plasminogen activator (tcu-PA) with stronger activity, which further activates plasminogen to produce large amounts of plasmin [45,49,50]. This interaction between scu-PA and plasminogen plays an important role in the initiation and acceleration of fibrinolysis. S-2444 is a specific substrate of tcu-PA that can catalyze its hydrolysis to p-nitroaniline [45,51]. Therefore, in our study, the uPA activity was monitored using S-2444 to effectively reflect the role of FGFC1 in the reaction system. It is noteworthy that the results of our fibrinolytic system showed that FGFC1 increased the fibrinolytic activity mediated by plasminogen and scu-PA and significantly shortened the time for the reaction system to reach the maximum reaction rate compared to the control group. These results further confirmed that FGFC1 could be used as a regulator of plasminogen or scu-PA to improve fibrinolytic activity and also indicated that the system constructed in vitro to simulate the physiological environment of the fibrinolytic system in vivo was successful.

Dissolved fibrin is essential to maintain the normal permeability of vascular walls and the flow state of blood. D-dimer is a specific marker of fibrinolysis and can be used to reflect intravascular thrombosis [52]. FITC-fibrin can provide a fluorescence signal for monitoring fibrinolysis [53,54]. Our study found that FGFC1 increased the fluorescence intensity and the concentration of D-dimer in FITC-fibrin lysate in a concentration-dependent manner. It is suggested that FGFC1 can effectively promote fibrinolysis in vitro. There is growing evidence that the structure of fibrin is an important determinant of thrombosis. A fibrin network composed of thin fibers is more resistant to fibrinolysis than one made of thick fibers [7]. We observed that the fibrin clot containing FGFC1 showed a loose and porous network structure using scanning electron microscopy and laser confocal microscopy. Compared to the control group, clots had lower fiber density and a larger fiber diameter and pores in the presence of FGFC1. This indicates that fibrin clots with FGFC1 can be dissolved more easily. These results showed that FGFC1 could effectively lysis fibrin in vitro, further confirming that FGFC1 could be used as a new small-molecule candidate drug for thrombolysis.

## 4. Materials and Methods

### 4.1. Materials

Urokinase chromogenic substrate S-2444 (Glu-Gly-Arg-pNA) and single-chain urokinase-type plasminogen activator (scu-PA) were purchased from BioMed (Shanghai, China) and Tasly Biopharmaceutical Co., Ltd, (Shanghai, China), respectively; human fibrinogen, plasminogen, thrombin, and fluorescein isothiocyanate isomer I (FITC) were purchased from Sigma-Aldrich (St Louis, MO, USA). Microplate readers were purchased from BioTek (Synergy 2, Winooski, VT, USA); scanning electron microscope was from Hitachi Inc. (SU5000, Tokyo, Japan); laser scanning confocal microscope was from Leica (Leica TCS SP8, Wetzlar, Germany).

**Figure 8:**
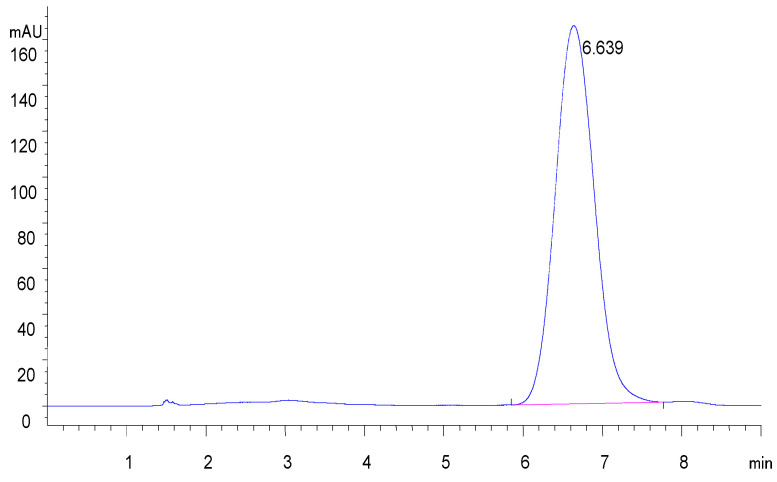
The chromatogram of FGFC1.

FGFC1, a chemical called 2,5-bis-[8-(4,8-dimethyl-nona-3,7-dienyl)-5,7-dihydroxy-8-methyl-3-keto-1,2,7,8-tetrahydro-6H-pyran [a]isoindol-2-yl]-pentanoic acid, was isolated from the metabolites of a rare marine fungi *Stachybotrys longispora* FG216 in our laboratory. Specifically, FGFC1 was obtained from the fermentation medium of *S. longispora* FG216 after precipitation, methanol separation, ethyl acetate extraction, and HPLC purification. The HPLC (high performance liquid chromatography) purity of the target compound was more than 98% (Figure 8) on an Inertsil PREP-ODS column (22.5 × 250 mm), which was developed at 40 °C with a gradient elution of acetonitrile and 0.1% trifluoroacetic acid at a rate of 1 mL/min [36,41].

### 4.2. Fibrinolytic Activity Measurements of FGFC1

The reaction system was made up of plasminogen (150 nM), S-2444 (1.2 mM), FGFC1(0, 0.4, 0.8, 1.6, 3.2, 6.4, 12.8, 25.6 μM), BSA (1%), scu-PA (15 nM), and Tris-Hcl buffer (50 mM, 100 mM NaCl, pH 7.4). The absorbance of this mixture system was determined at 37 °C for 180 min using a microplate reader. The fibrinolytic activity of FGFC1 was obtained from the absorbance–time curves. 

### 4.3. Preparation of Fluorescein Isothiocyanate (FITC)-Labeled Fibrin

A 30 mg amount of human fibrinogen was labeled with 3 mg FITC in 30 mL carbonate buffer (0.1 M, pH 9.0) and stirred at 4 °C overnight. The reaction was terminated by the addition of 0.15 M hydroxylamine and dialyzed in carbonate buffer (0.1 M, pH 9.0) at 4 °C overnight in the dark to remove unbound FITC molecules. FITC-fibrinogen was incubated with calcium chloride (0.1%) and thrombin (10 μ/mL) at room temperature for 2 h to form FITC-fibrin. After centrifugation at 3000 rpm for 10 min, the precipitate (FITC-fibrin) was collected and stored at −80 °C.

### 4.4. Determination of FITC-Fibrin Dissolution In Vitro

The reaction mixture contained Tris-Hcl buffer (50 mM, 100 mM NaCl, pH 7.4), BSA (1 %), FGFC1 (0, 0.4, 0.8, 1.6, 3.2, 6.4, 12.8 µM), plasminogen (0.15 µM), FITC-fibrin (saturated solution 5 µL), and scu-PA (15 nM). The reaction system was incubated at 37 °C for 20 min. After centrifugation at 3000 rpm for 10 min, 50 µL supernatant was collected, and its fluorescence intensity was then determined using a fluorescence microplate reader with an excitation wavelength of 495 nm and an emission wavelength of 525 nm to evaluate the fibrinolytic activity of FGFC1. In addition, we also used enzyme-linked immunosorbent assay (ELISA) to measure the d-dimer concentration in the solution of the above reaction system.

### 4.5. Scanning Electron Microscopy

Human fibrinogen (300 µM) was incubated with plasminogen (150 nM), scu-PA (15 nM), FGFC1 (0, 0.4, 0.8, 1.6, 3.2, 6.4, 12.8 µM), and thrombin and CaCl_2_ (final concentrations of 1 U/mL and 15 mM, respectively) in Tris-Hcl buffer at 37 °C for 50 min. Clots were washed with Tris-Hcl buffer 3 times and fixed at room temperature for 2 h in 2% glutaraldehyde. Clots were dehydrated with an acetone gradient and analyzed with a field-emission scanning electron microscope.

### 4.6. Confocal Microscopy

The reaction mixture system contained FGFC1 (0, 0.4, 0.8, 1.6, 3.2, 6.4, 12.8 uM), plasminogen (50 nM), human fibrinogen (30 uM), scu-PA (3 nM), FITC (100 ng/mL), Tris-Hcl buffer, and thrombin and CaCl_2_ (final concentrations of 0.6 U/mL and 8 mM, respectively) and was transferred into the channel of an uncoated ibidi slide and incubated at 37 °C for 60 min. Samples were visualized using a laser confocal microscope on an inverted microscope with 63× oil immersion objective lens, and images were 1024 pixels × 1024 pixels.

### 4.7. Statistical Analysis

Data were analyzed with GraphPad Prism 8 using the *t*-test. Data were expressed as mean ± standard deviation (SD) of 3 separate experiments. Two-sided *p* values less than 0.05 were regarded as statistically significant (* *p* < 0.05, ** *p* < 0.01).

## 5. Conclusions

Our results show that FGFC1 has good fibrinolytic activity in vitro. In addition, this is the first report that FGFC1 affects the structure of fibrin in the clot-dissolving process, causing it to have a lower density and a larger fiber diameter and pores and to be more sensitive to lysis. The results of this study are expected to contribute to supporting FGFC1 as a new candidate agent for thrombolytic therapy However, this study only evaluated the fibrinolytic effect of FGFC1 in vitro, so an in vivo and clinical study needs to be carried out to further confirm whether FGFC1 plays beneficial role in thrombolysis and to overcome the shortcomings.

## Data Availability

Not applicable.
